# Trifluridine/tipiracil overcomes the resistance of human gastric 5-fluorouracil-refractory cells with high thymidylate synthase expression

**DOI:** 10.18632/oncotarget.24412

**Published:** 2018-02-05

**Authors:** Kazuaki Matsuoka, Fumio Nakagawa, Takashi Kobunai, Teiji Takechi

**Affiliations:** ^1^ Translational Research Laboratory, Taiho Pharmaceutical Co., Ltd., Tokushima, Japan; ^2^ Applied Pharmacology Laboratory, Taiho Pharmaceutical Co., Ltd., Tokushima, Japan

**Keywords:** trifluridine/tipiracil, TAS-102, 5-FU resistant cells, gastric cancer

## Abstract

Trifluridine/tipiracil (FTD/TPI or TFTD, also known as TAS-102) is a combination of the antineoplastic thymidine analog, FTD, and thymidine phosphorylase inhibitor, TPI (molar ratio 1:0.5). FTD/TPI was approved in Japan, the United States, and the European Union for the treatment of unresectable advanced or recurrent colorectal cancer. We evaluated the *in vitro* and *in vivo* efficacy and mechanisms of action of FTD and FTD/TPI against 5-fluorouracil (5-FU)-resistant MKN45/5FU, MKN74/5FU, and KATOIII/5FU human gastric cancer cells overexpressing thymidylate synthase (TS) and their respective parent cell lines. MKN45/5FU and KATOIII/5FU cells were not cross-resistant to FTD, whereas MKN45/5FU cells were 3.7-fold more resistant than the parental cells *in vitro*. FTD was also incorporated into genomic DNA in a concentration-dependent manner in 5-FU-resistant and parental cells. Additionally, deoxyuridine monophosphate levels in MKN45/5FU cells after 24-h FTD treatment were 3.0-fold higher than those in parental cells, and FTD treatment for 72 h induced G2/M arrest in MKN45/5FU cells, unlike the S phase arrest in MKN45 cells. Thus, TS-overexpressing MKN45/5FU cells, but not MKN74/5FU and KATOIII/5FU cells, showed partial cross-resistance to FTD. However, FTD/TPI (administered orally twice a day) exhibited antitumor activity to the same extent in MKN45 and MKN45/5FU xenograft mouse models, overcoming *in vitro* cross-resistance to FTD. DNA incorporation rather than TS inhibition seems to be the main action of FTD under these *in vivo* conditions. Thus, FTD/TPI is a promising chemotherapeutic agent against gastric cancers recurring following 5-FU therapy.

## INTRODUCTION

Gastric cancer is the fourth leading cause of cancer-related deaths and accounts for approximately 750000 deaths each year worldwide [1]. Although significant survival benefits have been reported from 5-fluorouracil (5-FU) chemotherapy, many patients experience recurrence after several courses. Thus, inherent or acquired resistance to this drug is a major clinical issue.

Investigating the mechanisms of 5-FU resistance may accelerate the development of novel and effective chemotherapies against refractory cancers. Several 5-FU-resistant cell lines have been established by repeatedly exposing colorectal and gastric cancer cell lines to the drug. In these cell lines, thymidylate synthase (TS) protein levels are increased [2] or 5-FU incorporation into RNA is reduced as a result of decreased orotate phosphoribosyltransferase (OPRT) activity [3, 4]. As OPRT-mediated conversion of 5-FU to fluorouridine monophosphate is the major route of 5-FU activation, decreased OPRT activity will also decrease 5-FU incorporation into DNA and formation of fluorodeoxyuridine monophosphate (FdUMP), most likely leading to decreased inhibition of TS. To the best of our knowledge, no 5-FU-resistant gastric cancer cell lines, in which TS expression is up-regulated, have been established prior to our recent report [5]. As abundant expression of TS confers 5-FU resistance, these cells may be useful for modeling clinical responses and investigating mechanisms underlying resistance [6–8].

Trifluridine/tipiracil (FTD/TPI or TFTD, also known as TAS-102) is a combination of FTD and TPI at a molar ratio of 1:0.5. FTD, an antineoplastic thymidine analogue [9], is the active antitumor component of FTD/TPI, whose triphosphate form is incorporated into DNA in tumor cells [10–12]. TPI potently inhibits thymidine phosphorylase [13], an enzyme that degrades FTD, maintaining adequate concentrations of orally administered FTD in the plasma [13] and thereby potentiating FTD antitumor activity. Numerous clinical studies of FTD/TPI have been performed [14–17]. In an international, multicenter randomized double-blind phase III study (RECOURSE trial), FTD/TPI significantly improved overall and progression-free survival compared to that of placebo, and had a favorable safety profile in patients with metastatic colorectal cancer refractory to standard chemotherapies [18].

However, the effectiveness of FTD/TPI in 5-FU-resistant gastric tumors that overexpress TS remains to be established. In this study, we evaluated for the first time, *in vitro* and *in vivo*, the efficacy and mechanism of action of FTD and FTD/TPI against such cancers.

## RESULTS

### *In vitro* growth inhibition of 5-FU-resistant cells

**Figure 1:**
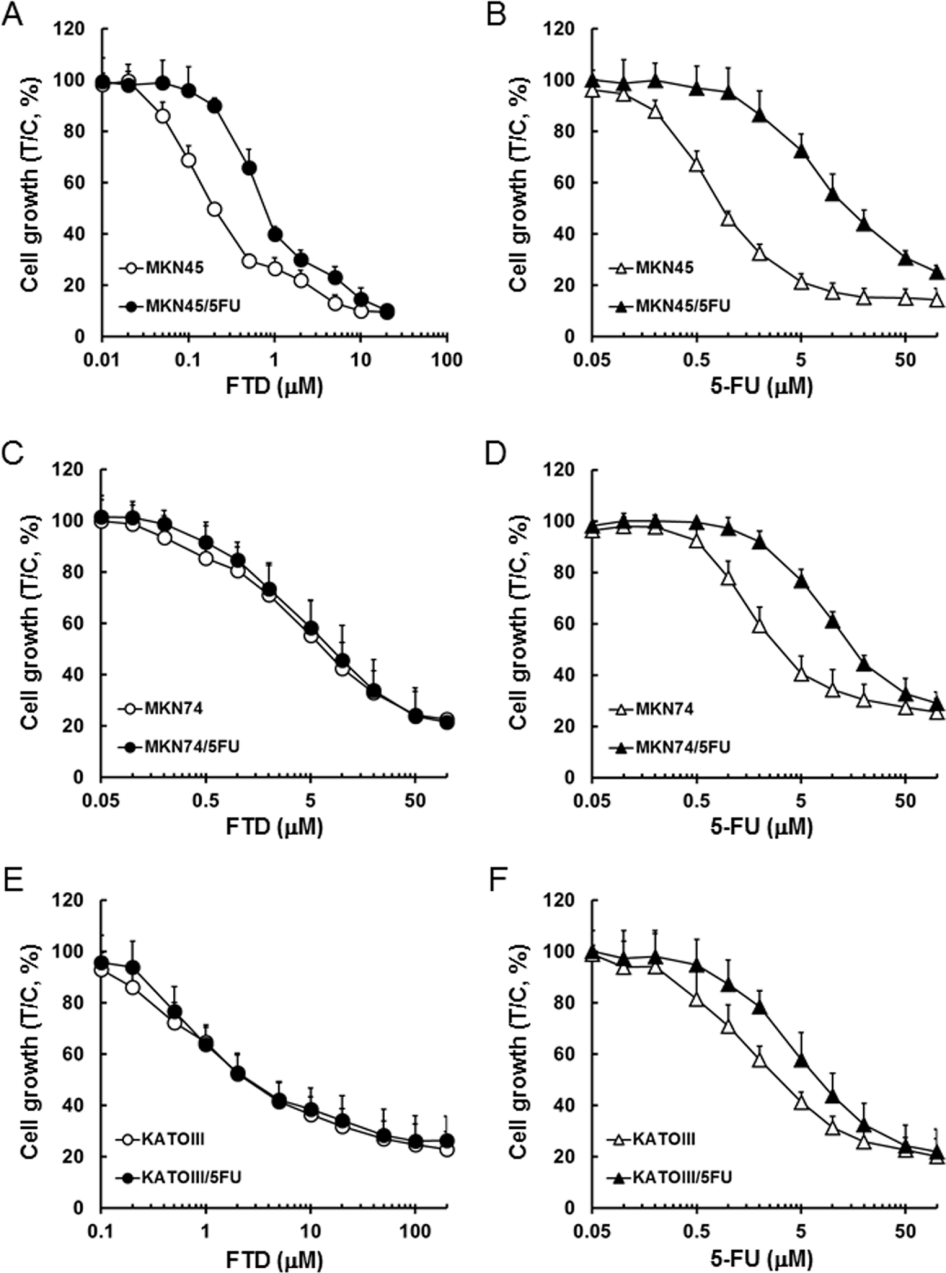
Inhibitory activity of FTD and 5-FU against cancer cells sensitive and resistant to 5-FU Cell lines were cultured with various concentrations of FTD and 5-FU for 72 h. Data are represented as the mean + SD of three independent experiments, normalized to the control. (**A**) and (**B**) MKN45 and MKN45/5FU, (**C**) and (**D**) MKN74 and MKN74/5FU, (**E**) and (**F**) KATOIII and KATOIII/5FU.

Growth inhibition effects of FTD and 5-FU in MKN45, MKN74, KATOIII, and their respective 5-FU-resistant cells are plotted in (Figure 1). IC_50_ values for FTD in MKN45, MKN45/5FU, MKN74, MKN74/5FU, KATOIII, and KATOIII/5FU were 0.23, 0.85, 6.0, 7.0, 2.7, and 2.7 µM, respectively, whereas the IC_50_ values for 5-FU in those cell lines were 0.93, 13.3, 3.2, 15.1, 2.9, and 7.1 µM, respectively. The resistant cell lines, MKN45/5FU, MKN74/5FU, and KATOIII/5FU, were 14.3-fold, 4.7-fold, and 2.4-fold more resistant to 5-FU, respectively, than their parental cells were. The MKN45/5FU cell line was 3.7-fold more resistant to FTD compared to that of the parental cells, whereas the resistance of MKN74/5FU and KATOIII/5FU cells was not increased.

### Antitumor activity of FTD/TPI and TPI against 5-FU-resistant xenografts

**Figure 2:**
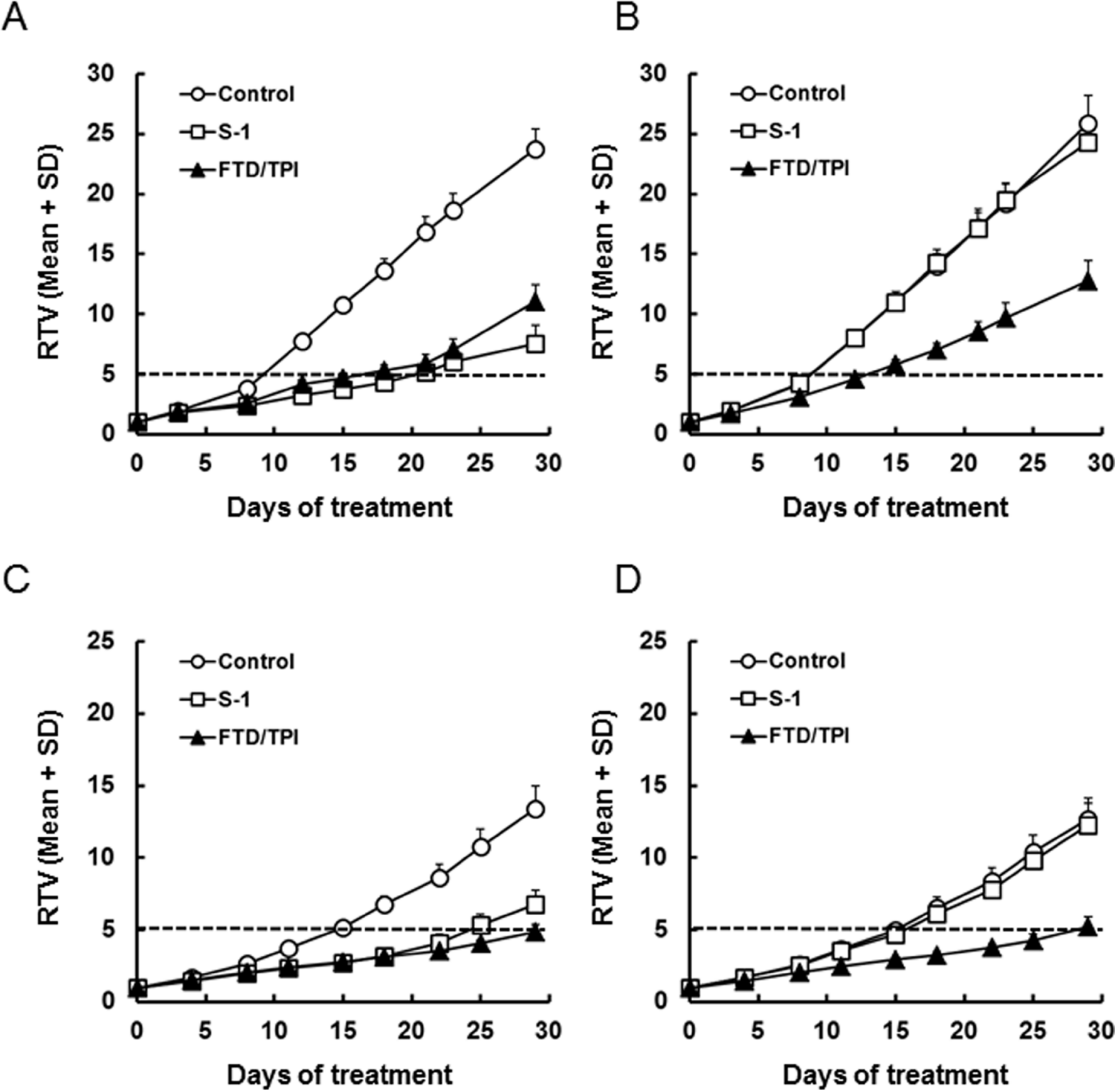
Relative tumor volume (RTV) of xenografted tumors after daily oral administration of FTD/TPI and S-1 Xenografted mice were randomized on day 0. FTD/TPI (150 mg/kg) and S-1 (10 mg/kg) were administered orally twice and once daily, respectively, from days 1 to 14. Data are represented as the mean + SD (*n* = 8). The horizontal dotted line indicates a relative tumor volume of 5. (**A**) MKN45, (**B**) MKN45/5FU, (**C**) MKN74, and (**D**) MKN74/5FU.

To determine whether MKN45/5FU cells are cross-resistant to FTD *in vivo*, we compared the activity of FTD/TPI and S-1 against xenografted MKN45 and MKN45/5FU. S-1 therapy is based on 5-FU and consists of tegafur, gimeracil, and oteracil at a molar ratio of 1:0.4:1 [19]. The relative tumor volume (RTV) of MKN45 xenografts is shown in (Figure 2A and Table 1). FTD/TPI exhibited significant antitumor activity on day 29 (*p* < 0.001), and significantly increased RTV5 (*p* < 0.001), the time required for a tumor to reach five times its initial volume [20]. FTD/TPI also showed significant activity (*p* < 0.001) against MKN45/5FU xenografts, whereas S-1 did not (Figure 2B and Table 1). Comparable antitumor activity of FTD/TPI against MKN45/5FU and MKN45 xenografts suggests that FTD/TPI overcame the resistance to 5-FU. In addition, as shown in Supplementary Figure 1A and 1B, body weight changes (BWC) were comparable in MKN45 and MKN45/5FU xenografted mice treated with FTD/TPI and S-1. We observed that untreated mice xenografted with MKN45 and MKN45/5FU lost more than 20% of their body weight as the tumor progressed, suggesting cancer-induced cachexia. In experiments with MKN74/5FU xenografts (Figure 2D and Table 2), antitumor activity of FTD/TPI was significant (*p* < 0.001) on day 29 and was comparable to activity against MKN74 xenografts (Figure 2C and Table 2), whereas the activity of S-1 was reduced in the MKN74/5FU xenografts. Overall, body weight decreases were not observed in mice xenografted with MKN74 and MKN74/5FU and treated with FTD/TPI and S-1 (Supplementary Figure 1C and 1D).

**Table 1:** Anti-tumor effects of FTD/TPI and S-1 in mice implanted with MKN45 and MKN45/5FU human gastric tumors

Group	Dose (mg/kg/day)	Treatment	*n*	RTV (mean ± SD)	TGI (%)	RTV5 (mean ± SD, days)
**MKN45**						
Control	-	Day1∼14, p.o., b.i.d.	8	23.75 ± 1.68	-	9.21 ± 0.19
FTD/TPI	150	Day1∼14, p.o., b.i.d.	8	11.02 ± 1.43^a)^	53.6	16.92 ± 2.33^b)^
S-1	10	Day1∼14, p.o., q.d.	8	7.52 ± 1.59^a)^	68.3	20.70 ± 2.59^b)^
**MKN45/5FU**						
Control	-	Day1∼14, p.o., b.i.d.	8	25.88 ± 2.35	-	8.44 ± 0.25
FTD/TPI	150	Day1∼14, p.o., b.i.d.	8	12.74 ± 1.74^a)^	50.8	12.99 ± 0.64^b)^
S-1	10	Day1∼14, p.o., q.d.	8	24.34 ± 1.42^c)^	6.0	8.80 ± 0.28^c)^

RTV: Relative tumor volume on day 29; TGI: Tumor growth-inhibition ratio on day 29; RTV5: time at which RTV reached 5.

^a)^*p* < 0.001 with Aspin-Welch’s *t*-test, compared to control.

^b)^*p* < 0.001 with log-rank test, compared to control.

^c)^not significant, compared to control.

**Table 2:** Anti-tumor effects of FTD/TPI and S-1 in mice implanted with MKN74 and MKN74/5FU human gastric tumors

Group	Dose (mg/kg/day)	Treatment	*n*	RTV (mean ± SD)	TGI (%)	RTV5 (mean ± SD, days)
**MKN74**						
Control	-	Day1∼14, p.o., b.i.d.	8	13.39 ± 1.61	-	14.76 ± 0.76
FTD/TPI	150	Day1∼14, p.o., b.i.d.	8	4.88 ± 0.45^a)^	63.6	28.31 ± 1.06^b)^
S-1	10	Day1∼14, p.o., q.d.	8	6.79 ± 0.97^a)^	49.3	23.28 ± 2.63^b)^
**MKN74/5FU**						
Control	-	Day1∼14, p.o., b.i.d.	8	12.66 ± 1.49	-	15.08 ± 0.80
FTD/TPI	150	Day1∼14, p.o., b.i.d.	8	5.21 ± 0.67^a)^	58.8	26.99 ± 1.86^b)^
S-1	10	Day1∼14, p.o., q.d.	8	12.25 ± 1.51^c)^	3.2	15.71 ± 0.92^c)^

RTV: Relative tumor volume on day 29; TGI: Tumor growth-inhibition ratio on day 29; RTV5: time at which RTV reached 5.

^a)^*p* < 0.001 with Aspin-Welch’s *t*-test, compared to control.

^b)^*p* < 0.001 with log-rank test, compared to control.

^c)^not significant, compared to control.

Next, we compared the antitumor activity of TPI alone against xenografted MKN45 and MKN45/5FU or MKN74 and MKN74/5FU. The RTV of MKN45 and MKN45/5FU xenografts is shown in Supplementary Figure 2A, 2B, and Supplementary Table 1. TPI alone did not exhibit antitumor activity on day 29 and did not increase RTV5 in the xenografts. Similar results were obtained in MKN74 and MKN74/5FU xenografts, as shown in Supplementary Figure 2C, 2D, and Supplementary Table 2.

### Incorporation of FTD into genomic DNA

**Figure 3:**
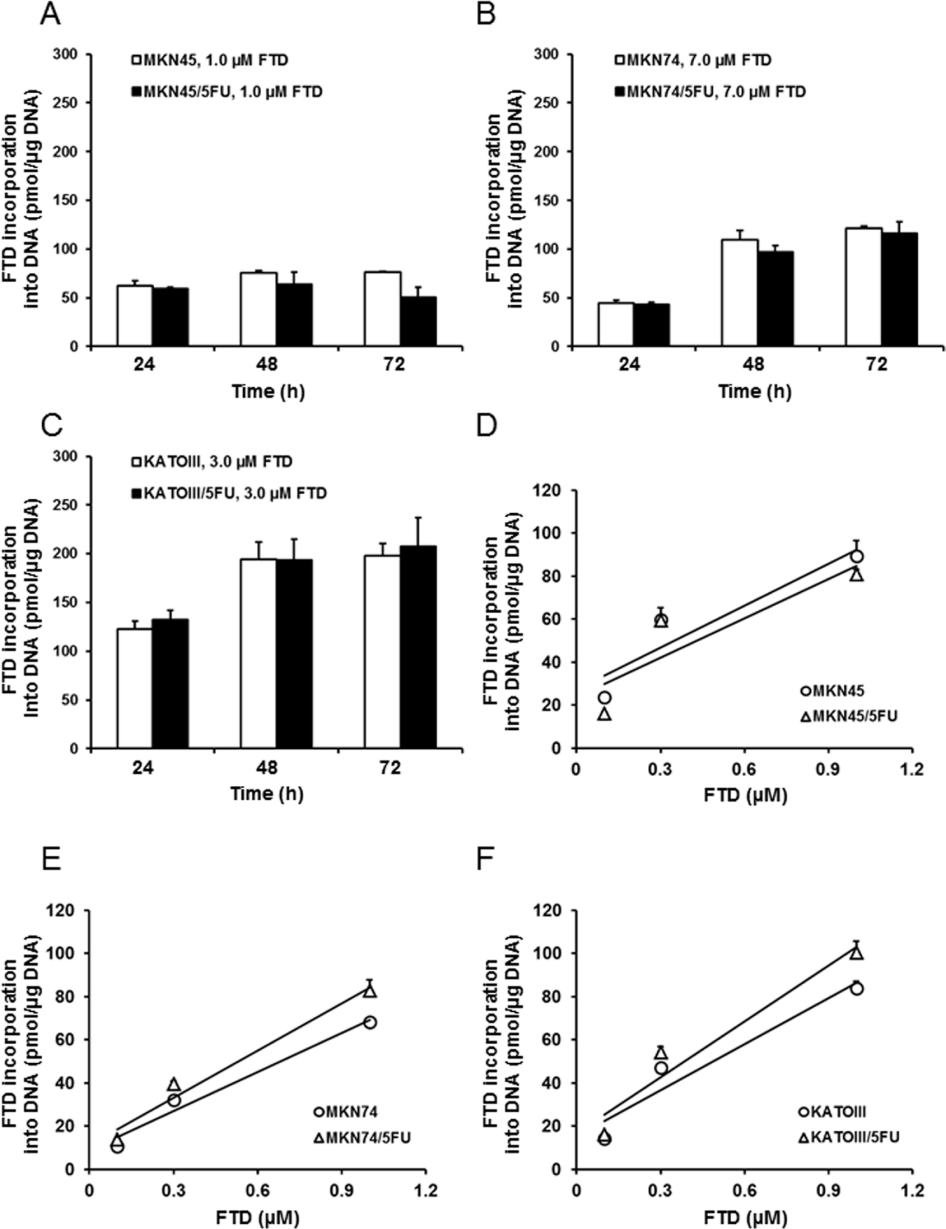
Incorporation of FTD into DNA DNA incorporation of FTD is shown as time-dependent (**A**–**C**) and dose-dependent (**D**–**F**) responses. (A) MKN45 and MKN45/5FU, (B) MKN74 and MKN74/5FU, and (C) KATOIII and KATOIII/5FU cells were treated with FTD for 24-72 h. Double-stranded DNA was extracted and FTD incorporation was determined by LC/MS/MS. Data are represented as the mean + SD (*n* = 3). FTD concentration was positively correlated with incorporation (Pearson correlation coefficients: (D) R^2^ = 0.85 (MKN45), 0.77 (MKN45/5FU). (E) R^2^ = 0.97 (MKN74), 0.97 (MKN74/5FU). (F) R^2^ = 0.92 (KATOIII), 0.93 (KATOIII/5FU)).

FTD has two mechanisms of action, DNA incorporation of its triphosphate form and TS inhibition by its monophosphate form. Therefore, we first examined whether DNA incorporation of FTD was different between parent and 5-FU-resistant cells. Based on IC_50_ values from the *in vitro* growth inhibition assay, incorporation was comparable between MKN45 and MKN45/5FU, MKN74 and MKN74/5FU, and KATOIII and KATOIII/5FU cells exposed to 1.0, 7.0, and 3.0 µM FTD, respectively (Figure 3). In both MKN45 and MKN45/5FU cells, incorporation of FTD into DNA seemed to reach a maximum after 24-h exposure, which was maintained from 48–72 h (Figure 3A), while FTD incorporation into DNA at 24 h was concentration-dependent (Figure 3D). The amount of DNA incorporation of FTD between MKN45 and MKN45/5FU cells was comparable. Since FTD reportedly induces cell cycle arrest [12, 21], these results suggest that FTD treatment at 1 µM for 24 h caused cell cycle delay, particularly in MKN45 and MKN45/5FU cells. As a result, further increases in FTD incorporation were not observed from 24–72 h. In contrast, the incorporation of FTD into DNA between MKN74 and MKN74/5FU and KATOIII and KATOIII/5FU cells reached a maximum after 24–48 h exposure, which was maintained at 72 h, with the amount of FTD incorporated into DNA comparable between the parental and 5-FU-resistant cells (Figure 3B and 3C). In addition, FTD was incorporated in a concentration-dependent manner in all of the cells (Figure 3E and 3F).

### Comparison of deoxyuridine monophosphate (dUMP) accumulation after treatment with FTD

**Figure 4:**
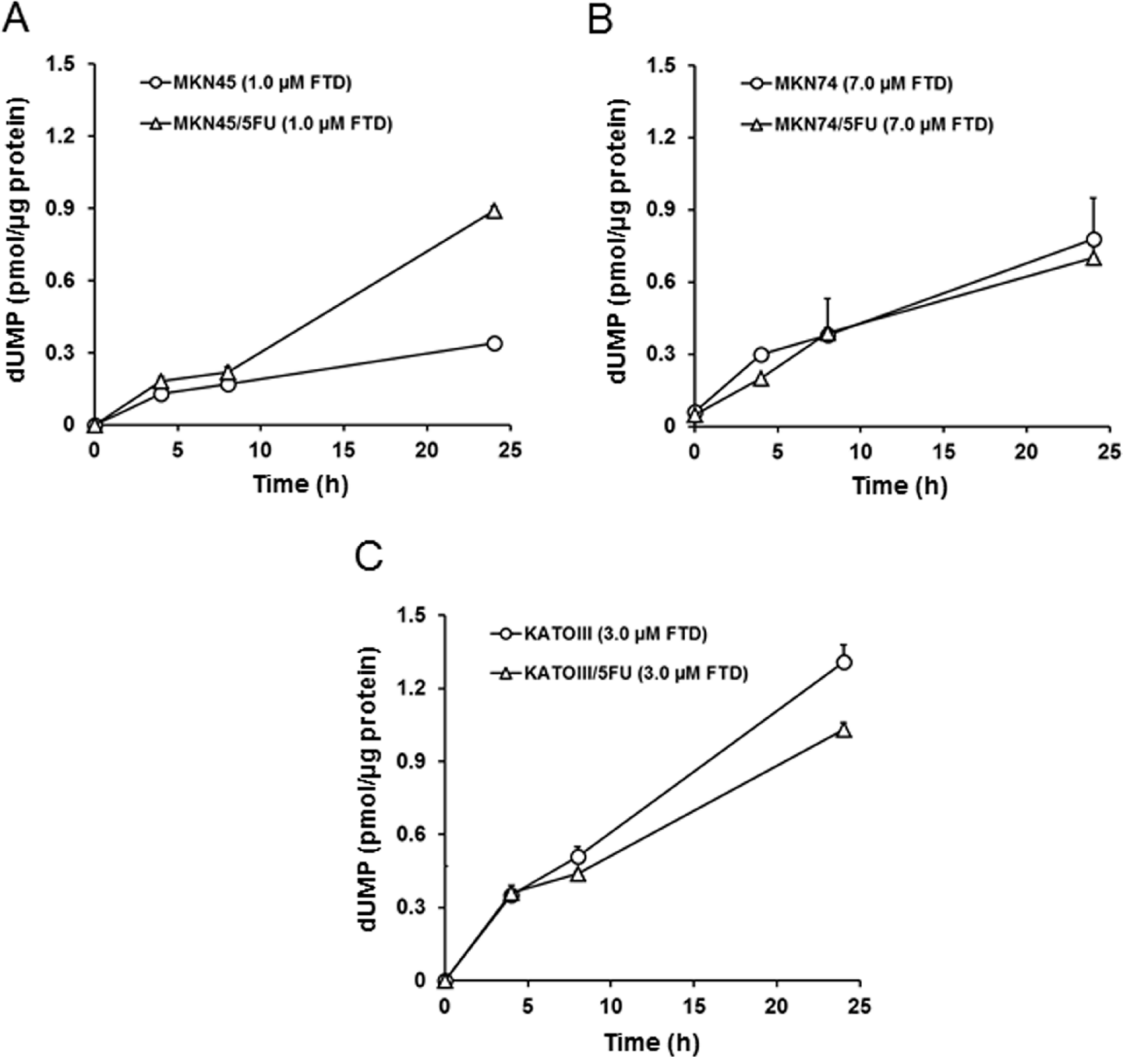
FTD-induced accumulation of dUMP The cells were exposed for 4, 8, and 24 h to indicated concentration of FTD. The cell dUMP levels were measured using LC/MS/MS. Data are represented as the mean + SD (*n* = 3). (**A**) MKN45 and MKN45/5FU, (**B**) MKN74 and MKN74/5FU, (**C**) KATOIII and KATOIII/5FU.

We next examined whether TS inhibition was different between the parent and 5-FU-resistant cells. In this experiment, we determined the amount of dUMP as an indicator of TS inhibition because it was previously reported that TS inhibition resulted in the accumulation of dUMP [22]. The FTD concentrations used were the same as those used for the evaluation of incorporation into genomic DNA. Although dUMP levels in MKN45 and MKN45/5FU cells increased time-dependently, dUMP levels in MKN45/5FU cells after 24-h treatment were approximately 3.0-fold higher than the levels in MKN45 (Figure 4A). dUMP levels in MKN74 and MKN74/5FU and KATOIII and KATOIII/5FU cells also increased time-dependently (Figure 4B and 4C), but no differences in dUMP levels between parent and 5-FU-resistant cells were observed.

### Cell cycle distribution

**Figure 5:**
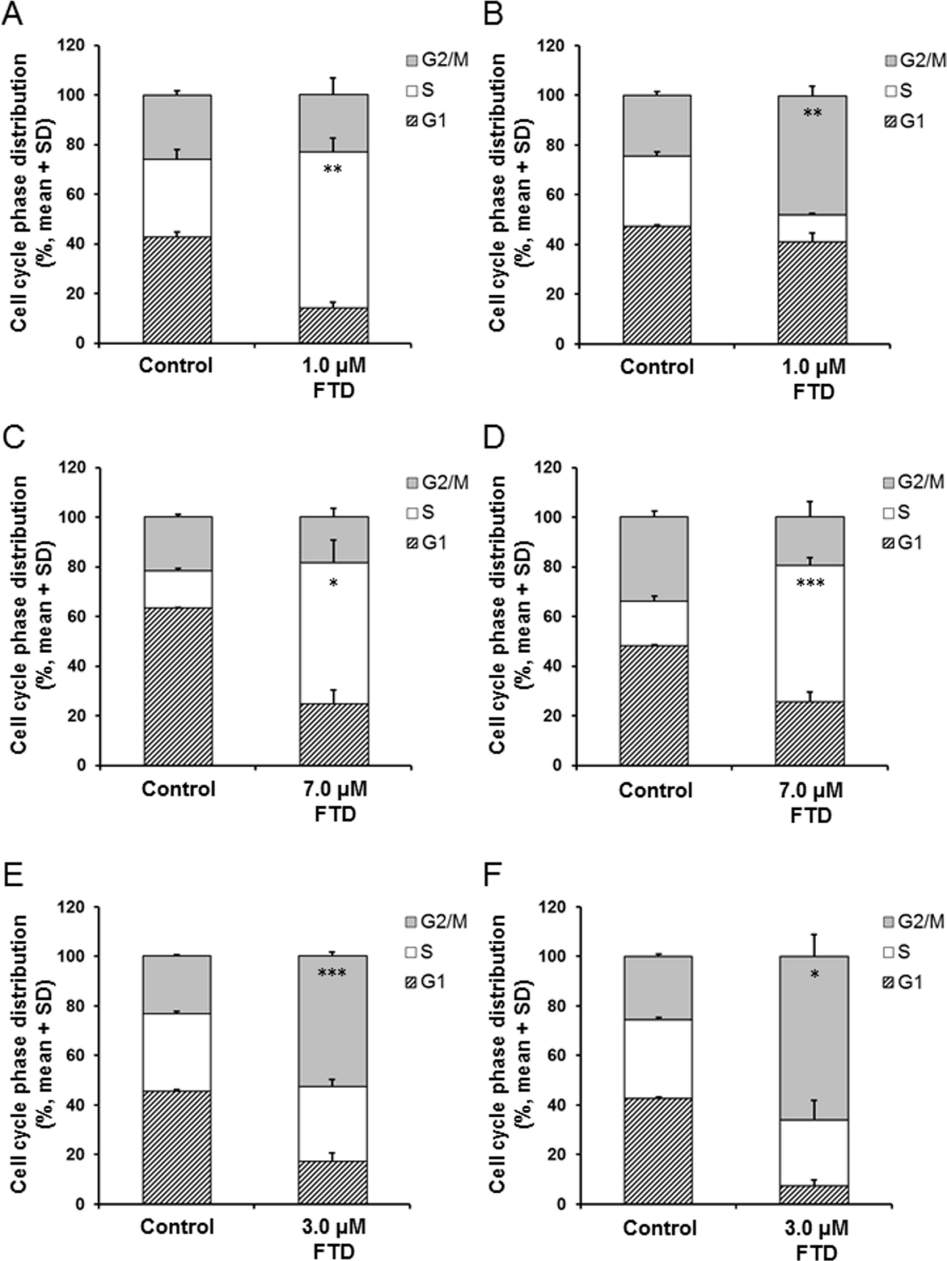
Effect of FTD on cell cycle distribution in parental and 5-FU resistant cells The cells were exposed for 72 h to indicated concentration of FTD and assayed using flow cytometry. Data are represented as the mean + SD of three independent experiments. ^*^p < 0.05; ^**^p < 0.01; ^***^p < 0.001. (**A**) and (**B**) MKN45 and MKN45/5FU, (**C**) and (**D**) MKN74 and MKN74/5FU, (**E**) and (**F**) KATOIII and KATOIII/5FU.

Cell cycle alterations by TS inhibition by 5-FU were previously reported [23, 24]. Thus, we examined the cell cycle distribution by flow cytometry after 72-h exposure of parental and 5-FU-resistant cells to FTD. Exposure to 1.0 µM FTD greatly altered cell cycle distribution in MKN45 and MKN45/5FU cells, and arrested the cells at the S and G2/M phases, respectively (Figure 5A and 5B). As TS expression in MKN45/5FU cells was 7.1-fold higher than that in MKN45 cells [5], TS inhibition with 1.0 µM FTD is thought to be insufficient in MKN45/5FU. Therefore, we speculate that the differences in cell cycle arrest points between MKN45 and MKN45/5FU cells are due to the difference in TS inhibition. In contrast, in MKN74 and MKN74/5FU cells, after exposure to 7.0 µM FTD, the cells were arrested in S phase (Figure 5C and 5D). After exposure to 3.0 µM FTD, KATOIII and KATOIII/5FU cells were arrested at the G2/M phase (Figure 5E and 5F). No differences in cell cycle distribution were observed between parental and corresponding 5-FU-resistant cells after exposure to FTD.

### Protein expression of ENT1 and TK1

FTD is transported into cells by the equilibrative nucleoside transporter 1 (ENT1) [23] and is converted to the monophosphate form, F_3_dTMP, by thymidine kinase 1 (TK1) [24, 25]. It was reported that both TK1 and ENT1 were involved in sensitivity to FTD [2]. Therefore, we compared TK1 and ENT1 protein expression in parental and 5-FU-resistant cells, as shown in Supplementary Figure 3. Since TK1 and ENT1 protein levels were comparable between parental and 5-FU-resistant cells, TK1 and ENT1 are not believed to be involved in the cross-resistance to FTD observed in MKN45/5FU cells.

## DISCUSSION

In this study, we, for the first time, evaluated the antitumor activity of FTD and FTD/TPI *in vitro* and *in vivo* against 5-FU-resistant gastric cancer cell lines that abundantly express TS. We found that these drugs were antitumorigenic *in vitro* and *in vivo*. Furthermore, FTD was incorporated into DNA in a concentration-dependent manner, and oral administration of FTD/TPI *in vivo* overcame the cross-resistance to FTD observed *in vitro*.

Two enzymes, OPRT and TS, were reported as the main factors responsible for 5-FU resistance in previously established cancer cell lines. Murakami *et al.* [3] reported that the enzymatic activity of OPRT was 0.58-fold lower in the 5-FU-resistant colorectal cancer cell line, DLD-1/5-FU, compared to that of the parental DLD-1 cells. Similarly, Inaba *et al.* [4] reported that OPRT activity was 0.31-fold lower in the 5-FU-resistant gastric cancer cell line, NUGC-3/5FU/L, than in the parental NUGC-3 cells. Reduced OPRT activity leads to decreased RNA incorporation, lower FdUMP formation, and decreased TS inhibition. Thus, reduced activity of OPRT is the main mechanism of resistance to 5-FU in both DLD-1/5-FU and NUGC-3/5FU/L cells. In contrast, Temmink *et al.* [2] reported that the 5-FU-resistant colorectal cancer cell line, H630-R10, with 12.0-fold higher TS levels than the parental H630, showed cross-resistance to FTD; the increased TS levels were suggested to cause FTD resistance. Importantly, we found that FTD overcame 5-FU resistance in MKN74/5FU and KATOIII/5FU cells *in vitro*, with MKN45/5FU cells exhibiting partial cross-resistance (Figure 1). To elucidate the cause of cross-resistance to FTD in MKN45/5FU cells, we investigated DNA incorporation of FTD and TS inhibition, which are the main mechanisms of action of FTD. There were no differences in FTD incorporation into DNA in all parental and 5-FU-resistant cells (Figure 3). However, dUMP levels in MKN45/5FU cells were approximately 3.0-fold higher than the levels in MKN45 cells at 24-h treatment with FTD, while no differences in dUMP levels between MKN74 and MKN74/5FU cells, and KATOIII and KATOIII/5FU cells were observed (Figure 4). We noted that TS expression in MKN45/5FU cells was the highest in all of the cells tested in this study [5]. We also investigated cell cycle distribution. After the treatment with FTD for 72 h, MKN45/5FU cells were arrested in G2/M phase, while MKN45 cells were arrested in S phase. Conversely, no differences in cell cycle accumulation between MKN74 and MKN74/5FU, or KATOIII and KATOIII/5FU were observed. Collectively, since there were no differences in FTD incorporation into DNA in all parental and corresponding 5-FU-resistant cells, DNA incorporation of FTD was not considered the cause of cross-resistance to FTD. Indeed, the main mechanism of action of FTD has been reported to be TS inhibition under continuous treatment *in vitro* [11, 25]. MKN74, KATOIII, and their corresponding 5-FU-resistant cells did not show cross-resistance to FTD, because the baseline TS expression between parental and 5-FU-resistant cells was low, and TS was fully inhibited in both cells. In contrast, since baseline TS expression in MKN45/5FU is reportedly high [5], we speculated that F_3_dTMP could not fully inhibit TS. As a result, differences in cell cycle accumulation of MKN45 and MKN45/5FU cells were expected. Thus, upregulating TS may lead to resistance to FTD under continuous treatment conditions.

**Figure 6:**
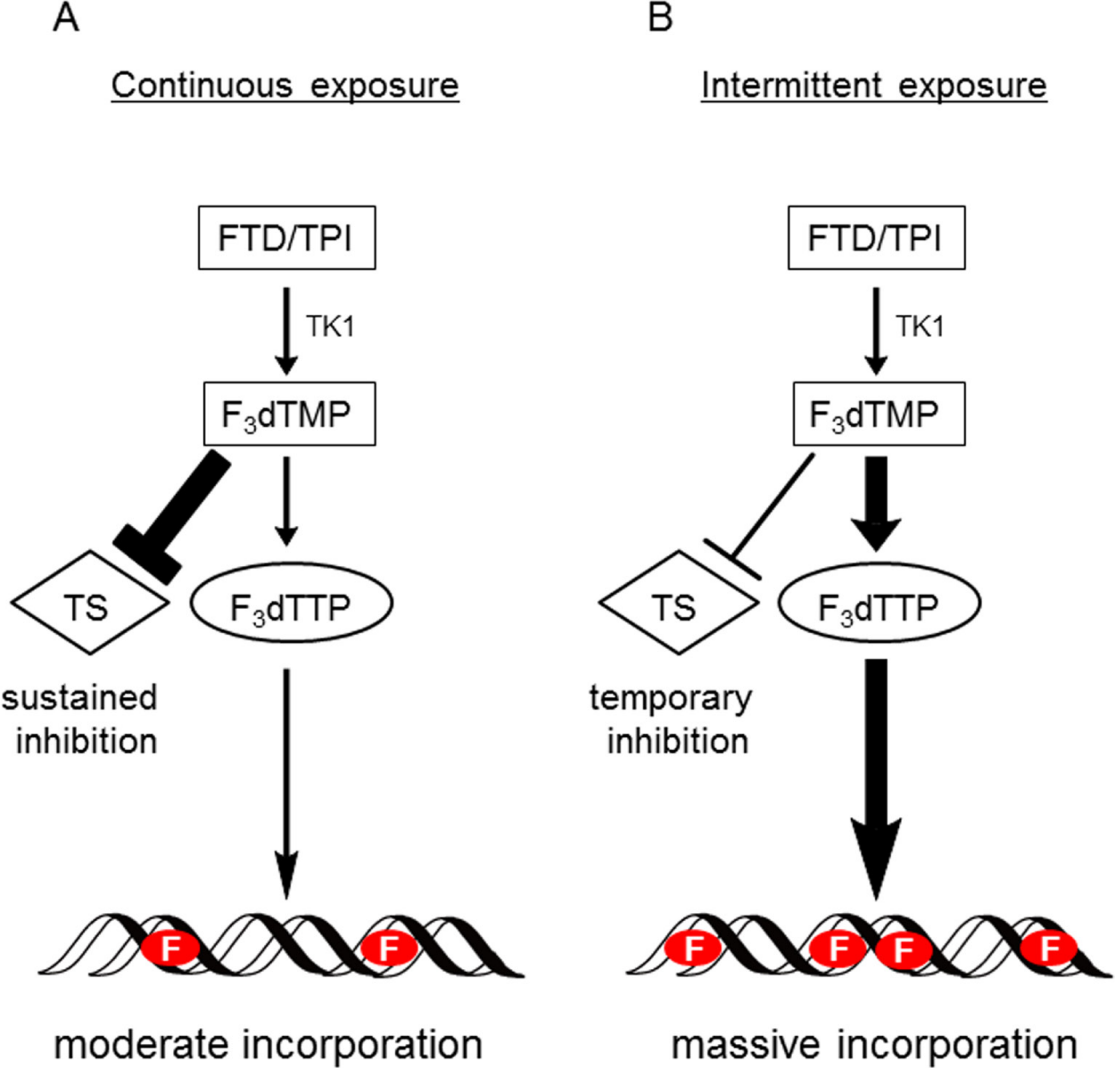
Hypothetical model of action of FTD with continuous exposure or intermittent exposure (**A**) When tumor cells are continuously exposed to FTD, such as during *in vitro* growth inhibition assay, TS inhibition significantly affects cytotoxicity. Therefore, it is presumed that MKN45/5FU cells with high TS expression show partial cross-resistance to FTD. (**B**) When FTD/TPI is administered orally twice a day (i.e., intermittent exposure), since plasma concentrations of FTD decrease between doses, TS inhibition recovers between doses. It is presumed that DNA incorporation of FTD predominantly induces antitumor effects, since FTD is incorporated into DNA in a concentration-dependent manner under this oral administration condition. Therefore, the cross-resistance to FTD was overcome even in the MKN45/5FU cells with high TS expression. F indicates FTD.

In contrast to the *in vitro* results, FTD/TPI was antitumorigenic against xenograft models of MKN45 and 5-FU-resistant MKN45/5FU cells (Figure 2). The antitumor activity of FTD/TPI was previously reported to depend on, and is positively correlated with, the amount of FTD incorporated into DNA [11]. Furthermore, it was reported that FTD incorporation into DNA, not inhibition of TS, is the major mechanism of FTD/TPI efficacy when FTD/TPI is administered orally twice a day as prescribed in the clinic [11]. Because TS inhibition by FTD is reversible [25], the inhibitory effect is thought to wane when plasma concentrations of FTD decrease between doses. Thus, we speculated that FTD was incorporated into DNA in the xenografted MKN45/5FU and MKN45 cells when FTD/TPI was administered orally twice daily, and that cross-resistance to FTD was overcome even in the MKN45/5FU cells with high TS expression. Summarizing our experimental data, we have generated a hypothetical model of the action of FTD in clinical use, which is shown in (Figure 6). It was reported that 5-FU treatment in patients initially decreases TS levels, but this is followed by TS induction [7]. We speculate that FTD/TPI can be effective against tumors with high TS induced by 5-FU treatment, because the antitumor activity of FTD/TPI is not dependent on the expression of TS. In the RECOURSE trial, since patients treated with 5-FU-based chemotherapies as front-line therapy were enrolled, TS expression might have been induced in the tumor tissue. However, FTD/TPI could be effective against these patients because this drug combination has a unique mechanism of action (i.e., DNA dysfunction), and is effective even against tumors with high TS levels.

Together, these data suggest that FTD is generally effective against 5-FU-resistant cells, incorporates into DNA, and ultimately induces cell cycle arrest at different phases in different cell lines. However, these results are based on only three cell lines, and further studies are needed to elucidate the mechanisms of FTD activity.

In conclusion, our findings suggest that FTD/TPI is a promising chemotherapeutic agent against gastric cancers overexpressing TS and recurring after 5-FU chemotherapy. A phase III study of FTD/TPI in patients with metastatic gastric cancer refractory to standard treatments is ongoing (NCT02500043), and we expect that its outcome will be highly informative.

## MATERIALS AND METHODS

### Ethics statement

All animal studies were performed according to the guidelines and with the approval of the Institutional Animal Care and Use Committee of Taiho Pharmaceutical Co., Ltd.

### Chemicals and reagents

FTD was obtained from Yuki Gosei Kogyo, Co., Ltd. (Tokyo, Japan), whereas 5-chloro-6-[(2-iminopyrrolidin-1-yl)methyl]pyrimidine-2,4-(1*H*,3*H*)-dione monohydrochloride (TPI), tegafur, gimeracil, and oteracil were obtained from Taiho Pharmaceutical (Tokyo, Japan). 5-FU and dimethylsulfoxide were purchased from Wako Pure Chemical Co., Ltd. (Osaka, Japan), whereas hydroxypropyl methylcellulose (HPMC) was procured from Shin-Etsu Chemical Co., Ltd. (Tokyo, Japan).

### Preparation of drugs

FTD/TPI was prepared by dissolving FTD and TPI in 0.5% HPMC at a molar ratio of 1:0.5. The doses administered are expressed as FTD. Orally administered S-1 suspension was prepared by dissolving tegafur, gimeracil, and oteracil in 0.5% HPMC at a molar ratio of 1:0.4:1, and the doses administered are expressed as tegafur.

### Cell lines

MKN45 cells were obtained from Health Science Research Resource Bank (Tokyo, Japan), whereas MKN74 and KATOIII cells were obtained from RIKEN BRC Cell Bank (Ibaraki, Japan). 5-FU-resistant cell lines MKN45/5FU, MKN74/5FU, and KATOIII/5FU were established after long-term culture in the presence of 5-FU, as described previously [5]. The cells were cultured at 37° C in a humidified atmosphere of 95% air and 5% CO_2_ in RPMI 1640 media supplemented with 10% fetal bovine serum, 100 U/mL penicillin, and 100 μg/mL streptomycin. These cells were authenticated in 2014 by analysis of short tandem repeats.

### Cell viability

Cell viability was determined by crystal violet staining [26]. Briefly, the cells were cultured in 96-well microplates for 16 h and exposed to various concentrations of FTD or 5-FU for 72 h. The cells were fixed with 2% glutaraldehyde for 20 min, stained for 20 min with 0.05% crystal violet in 20% methanol, and rinsed with tap water. Plates were dried on paper for 1 h and contents of wells were dissolved in 100 µL of a 1:1 mixture of ethanol and 0.1 M sodium dihydrogen phosphate. Absorbance was determined on a VersaMax microplate reader (Molecular Devices, Sunnyvale, CA, USA) at 540 nm, and cell viability is reported as the percentage of viable cells compared to control treated with 0.1% dimethylsulfoxide. IC_50_ values, i.e., the concentrations at which growth was inhibited by 50%, were calculated using XLfit software (ID Business Solutions, Guildford, UK).

### *In vivo* anti-tumor activity

Male nude mice (BALB/cA Jcl-*nu*/*nu*, 5-week old) were purchased from CLEA Japan Inc. (Tokyo, Japan), housed under specific pathogen-free conditions, and provided food and water *ad libitum*. After one week in quarantine, the mice were implanted subcutaneously (4 × 10^6^ cells/mouse) with MKN45, MKN45/5FU, MKN74, or MKN74/5FU cells suspended in saline. To evaluate antitumor activity, the mice were randomized into treatment groups, although care was taken to ensure that treatment groups contained a similar range of initial tumor volumes (150 to 200 mm^3^ at day 0). FTD/TPI was administered orally (150 mg/kg/day) twice a day from day 1 to 14, with approximately 6 h between doses [10, 13]. S-1 was administered orally (10 mg/kg/day) once a day from day 1 to 14 [27, 28]. Control animals were dosed with 0.5% HPMC (10 mL/kg) according to similar schedules. RTV was calculated using the following formula: RTV = (tumor volume on measured day)/(tumor volume on day 0). On day 29, the tumor growth inhibition ratio (TGI,%) was calculated using the following formula: TGI (%) = [1–(RTV in experimental group)/(RTV in control group)] × 100 (%). Antitumor activity was also evaluated based on RTV5, which is the time required for RTV to reach 5. To estimate RTV5, RTV was plotted for each mouse, and the date on which RTV reached 5 was determined by linear regression [29]. Differences in mean RTV on day 29 were tested for statistical significance using Aspin-Welch’s *t*-test. The statistical analysis of RTV5 was evaluated using the log-rank test [20]. Finally, toxicity was evaluated based on BWC, which was calculated using the following formula: BWC (%) = [(body weight on measured day – body weight on day 0)/body weight on day 0] × 100 (%).

### Immunoblotting

Cell pellets were lysed in RIPA buffer (Thermo Fisher Scientific, Waltham, MA, USA) containing protease and phosphatase inhibitors cocktails (Nacalai Tesque, Kyoto, Japan), and incubated for 30 minutes on ice. The supernatant was cleared by centrifugation at 15000 **×** g and 5° C for 15 minutes. Because ENT1 is localized to the cell membrane [30], lysates were also prepared using Mem-PER Plus Membrane Protein Extraction Kit (Thermo Fisher Scientific) according to the manufacturer’s instructions. Protein concentration was determined using the BCA Protein Assay Kit (Thermo Fisher Scientific). Equal amounts of protein (10 µg/lane for TK1 and 0.2 µg/lane for ENT1) were resolved by SDS-PAGE and analyzed by western blot using antibodies against TK1 (ab76495, Abcam, Cambridge, MA, USA) and ENT1 (sc377283, Santa Cruz Biotechnology, Santa Cruz, CA, USA). Blots were visualized on an ImageQuant LAS 3000 Mini system (GE Healthcare UK Ltd.).

### FTD incorporation into DNA

DNA was extracted from cells treated with FTD using NucleoSpin Blood L (Takara Bio Inc., Tokyo, Japan), following the manufacturer’s protocol. DNA concentration was determined using Qubit dsDNA Broad-Range Assay Kits (Thermo Fisher Scientific) and samples were diluted to 10 μg/mL with distilled water and degraded to nucleosides using a modified published method [11]. Briefly, a 300-μL reaction mixture consisting of 100 mM Tris-HCl (pH 7.0), 50 mM NaCl, 2.5 mM CaCl_2_, 10 mM MgCl_2_, 1 U of DNase I (Takara Bio Inc.), 40 μg of phosphodiesterase I (Sigma-Aldrich, St. Louis, MO, USA), 2 U of alkaline phosphatase (Takara Bio Inc.), and 2 μg of extracted DNA was incubated at 37° C for 2 h. After the reaction, 30 μL of 4.2 N perchloric acid was added to each reaction mixture and the mixture was incubated on ice for 10 minutes. The samples were neutralized with K_2_HPO_4_ (2 M, 90 μL) and centrifuged at 15000 **×** g and 5° C for 30 minutes. The obtained supernatant was analyzed by LC/MS/MS. Samples for LC/MS/MS were prepared by mixing 100 μL of sample, 10 μL of water, 50 μL of 1 M hydrochloric acid, and 20 μL of internal standard working solution. The mixture was extracted with 1 mL of methyl *t*-butyl ether and centrifuged at 15000 **×** g and 5° C for 5 min. The supernatant was dried under nitrogen at 40° C and the residue was reconstituted with 0.1 mL of 0.1% acetic acid (A) and acetonitrile (B) (75:25, v/v of A:B). Aliquots (5 μL) of reconstituted samples were analyzed on an API 4000 LC/MS/MS system (AB Sciex, Foster City, CA, USA). Incorporation (pmol) of FTD into DNA was represented as the amount of FTD per µg of DNA.

### Determination of dUMP levels in cells after treatment with FTD

dUMP levels were determined using a modified published method [31]. Methanol (500 µL) containing 20 µg/mL 5-chlorouracil was added to cell pellets treated with FTD for 4, 8, and 24 h, vortexed vigorously, and centrifuged at 15000 × *g* and 5° C for 5 min. The supernatant was dried under nitrogen at 40° C and the residue was reconstituted with 0.1 mL of 10 mM ammonium formate (A) and acetonitrile (B) (95:5, v/v of A:B). Aliquots (5 μL) of reconstituted samples were analyzed on an API 4000 LC/MS/MS system (AB Sciex). Total protein content of cell pellets was determined using the BCA Protein Assay Kit (Thermo Fisher Scientific). The amount of dUMP was expressed in pmol per µg protein.

### Cell cycle analysis

MKN45, MKN45/5FU, KATOIII, KATOIII/5FU, MKN74, and MKN74/5FU cells cultured in the presence of FTD or 5-FU for 72 h were fixed with 70% ice-cold ethanol. To determine DNA content, the cells were stained with 5 µg/mL propidium iodide supplemented with 10 µg/mL RNase A. Samples were analyzed using a BD Accuri C6 flow cytometer (BD Biosciences, San Jose, CA, USA) and FlowJo software (Tree Star, Inc., Ashland, OR, USA). Accumulation of cells in cell cycle phases was tested for statistical significance using Aspin-Welch’s *t*-test.

## SUPPLEMENTARY MATERIALS FIGURES AND TABLES


