# Supplementary material for: Trifluridine/tipiracil overcomes the resistance of human gastric 5-fluorouracil-refractory cells with high thymidylate synthase expression
Source: Oncotarget. 2018 Feb 5;9(17):13438–50. doi: 10.18632/oncotarget.24412 (PMC5862589; doi:10.18632/oncotarget.24412)
Supplement: Supplementary file 1 [file oncotarget-09-13438-s001.pdf]

Trifluridine/tipiracil overcomes the resistance of human gastric 5-fluorouracil-refractory cells with high thymidylate synthase expression

SUPPLEMENTARY MATERIALS

Supplementary Table 1: Anti-tumor effects of TPI in mice implanted with MKN45 and MKN45/5FU human gastric tumors

Group	Dose (mg/kg/day)	Treatment	*n*	RTV (mean ± SD)	TGI (%)	RTV5 (mean ± SD, days)
**MKN45**						
Control	-	Day1~14, p.o., b.i.d.	8	22.78 ± 0.75	-	9.29 ± 0.26
TPI	70	Day1~14, p.o., b.i.d.	8	24.14 ± 0.92[c)]	–6.0	9.10 ± 0.30[c)]
**MKN45/5FU**						
Control	-	Day1~14, p.o., b.i.d.	8	23.82 ± 2.87	-	9.10 ± 0.56
TPI	70	Day1~14, p.o., b.i.d.	8	24.53 ± 3.49[c)]	–3.0	9.16 ± 0.40[c)]

RTV: Relative tumor volume on day 29; TGI: Tumor growth-inhibition ratio on day 29; RTV5: time at which RTV reached 5.
[c)]not significant, compared to control.

Supplementary Table 2: Anti-tumor effects of TPI in mice implanted with MKN74 and MKN74/5FU human gastric tumors

Group	Dose (mg/kg/day)	Treatment	*n*	RTV (mean ± SD)	TGI (%)	RTV5 (mean ± SD, days)
**MKN74**						
Control	-	Day1~14, p.o., b.i.d.	8	15.05 ± 2.73	-	13.58 ± 0.85
TPI	70	Day1~14, p.o., b.i.d.	8	14.43 ± 0.96[c)]	4.1	13.87 ± 0.30[c)]
**MKN74/5FU**						
Control	-	Day1~14, p.o., b.i.d.	8	17.86 ± 2.22	-	12.60 ± 0.41
TPI	70	Day1~14, p.o., b.i.d.	8	17.92 ± 1.31[c)]	–0.3	12.52 ± 0.53[c)]

RTV: Relative tumor volume on day 29; TGI: Tumor growth-inhibition ratio on day 29; RTV5: time at which RTV reached 5.
[c)]not significant, compared to control.

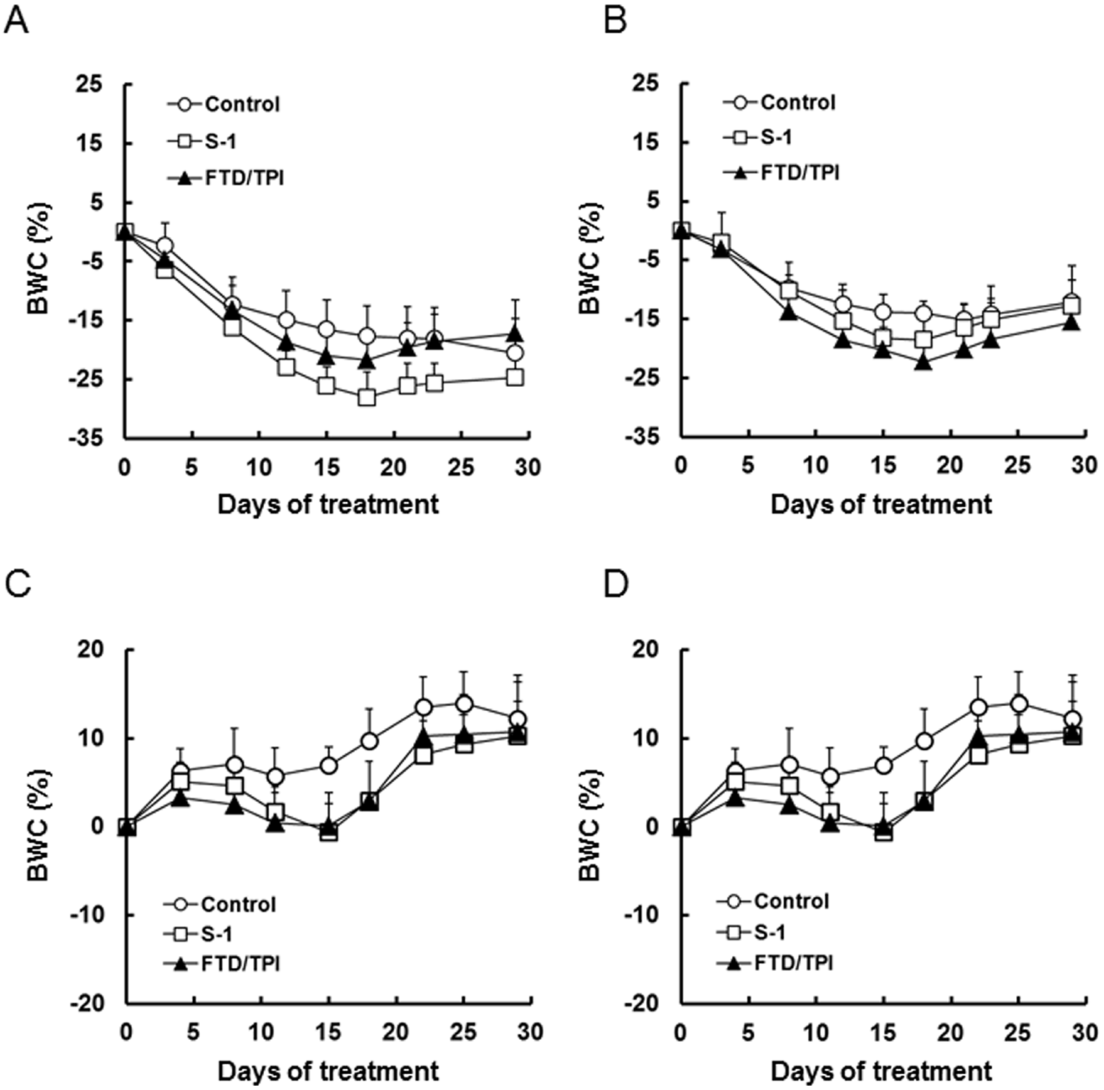

Supplementary Figure 1: Body weight change (BWC) of xenografted mice after daily oral administration of FTD/TPI and S-1. Xenografted mice were randomized on day 0. FTD/TPI (150 mg/kg) and S-1 (10 mg/kg) were administered orally twice and once daily, respectively, from days 1 to 14. Data are represented as the mean + SD (*n* = 8). (**A**) MKN45, (**B**) MKN45/5FU, (**C**) MKN74, and (**D**) MKN74/5FU.

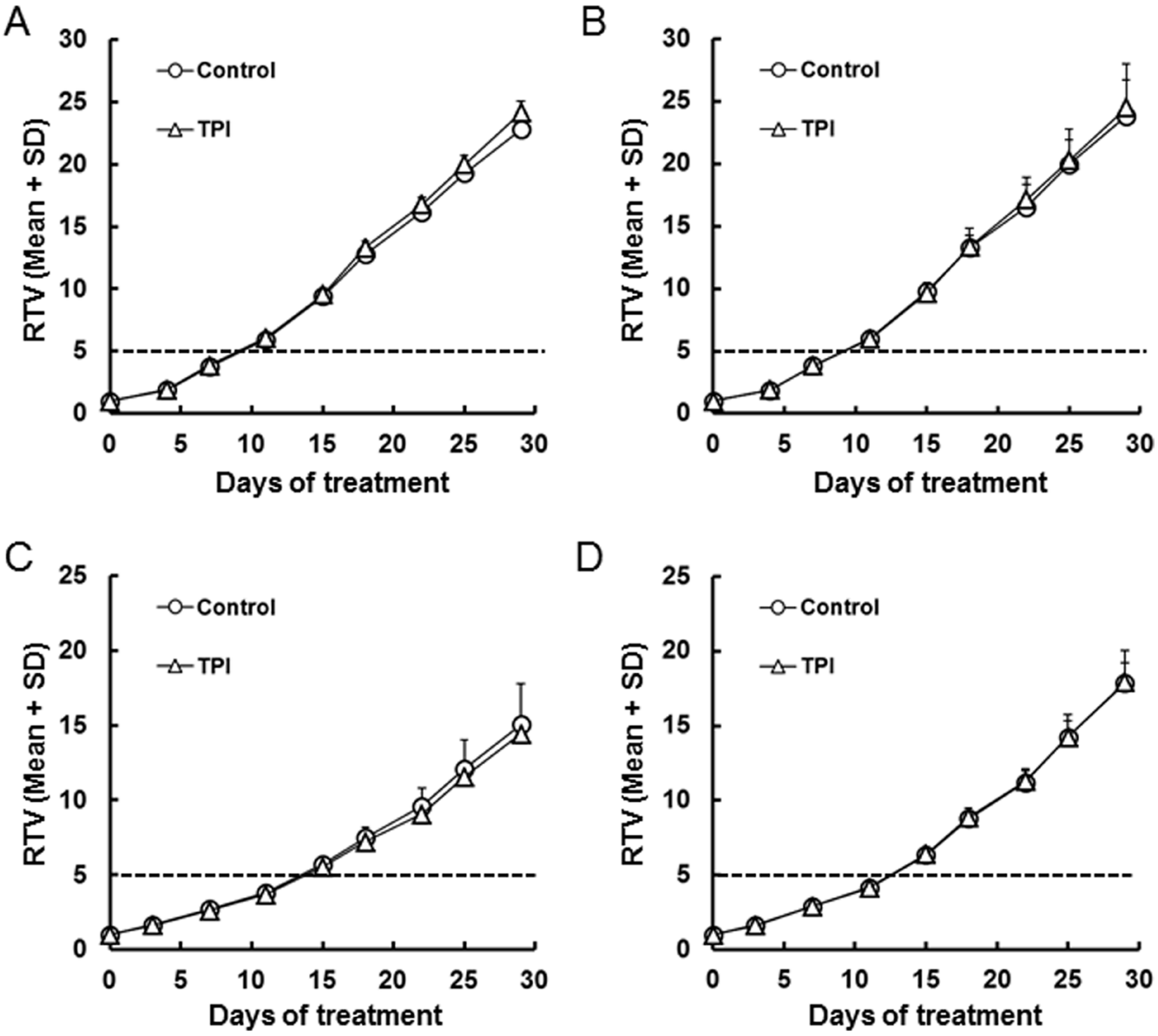

Supplementary Figure 2: Relative tumor volume (RTV) of xenografted tumors after daily oral administration of TPI. Xenografted mice were randomized on day 0. TPI (70 mg/kg) was administered orally twice daily from days 1 to 14. Data are represented as the mean + SD (*n* = 8). The horizontal dotted line indicates a relative tumor volume of 5. (**A**) MKN45, (**B**) MKN45/5FU, (**C**) MKN74, and (**D**) MKN74/5FU.

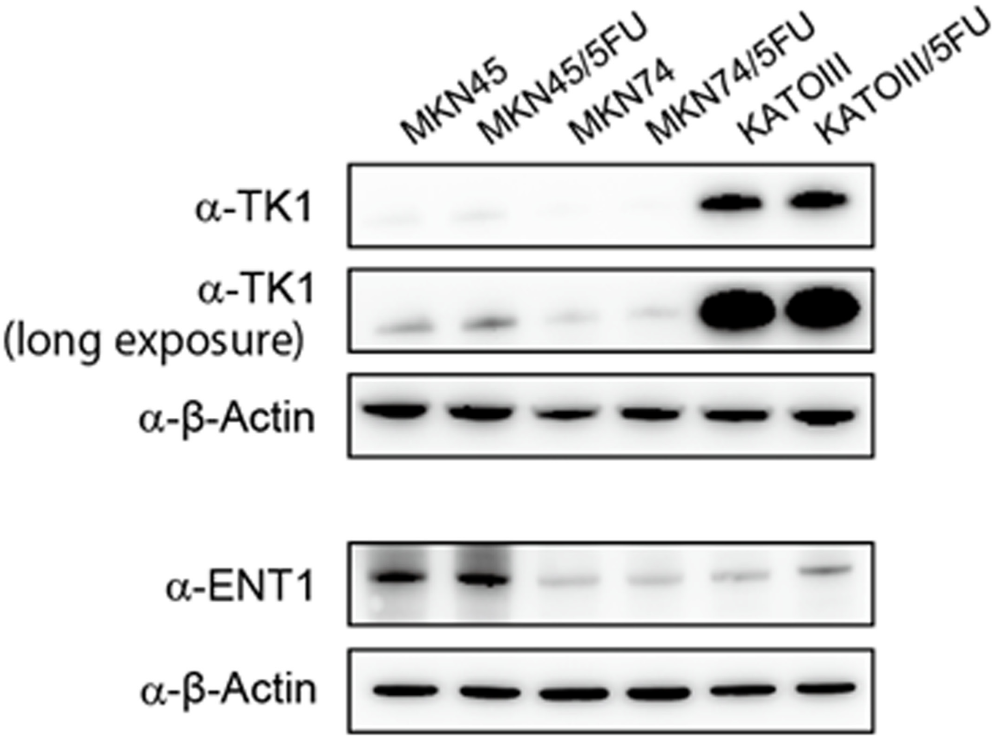

Supplementary Figure 3: Protein expression of TK1 and ENT1 in parental and 5-FU resistant cells. Expression of TK1 and ENT1 was determined by immunoblotting.
